# The complete mitochondrial genome of Xizicus (Haploxizicus) maculatus revealed by Next-Generation Sequencing and phylogenetic implication (Orthoptera, Meconematinae)

**DOI:** 10.3897/zookeys.773.24156

**Published:** 2018-07-09

**Authors:** Mao Shaoli, Yuan Hao, Lu Chao, Zhou Yafu, Shi Fuming, Wang Yuchao

**Affiliations:** 1 Xi’an Botanical Garden of Shaanxi Province/Institute of Botany of Shaanxi Province, Xi’an 710061, China; 2 College of Life Sciences, Shaanxi Normal University, Xi’an 710062, China; 3 College of Life Sciences,; 4 Hebei University, Baoding, Hebei 071002, China

**Keywords:** mitochondrial genome, Next-Generation Sequencing, phylogenetic relationship, Xizicus (Haploxizicus) maculatus

## Abstract

*Xizicus* Gorochov, 1993, the quiet-calling katydid, is a diverse genus with 68 species in world, which includes more than 45 species in China, has undergone numerous taxonomic revisions with contradicting conclusions. In this study the complete mitochondrial genome of Xizicus (Haploxizicus) maculatus collected from Hainan for the first time was sequenced using the Next-Generation Sequencing (NGS) technology. The length of whole mitogenome is 16,358 bp and contains the typical gene arrangement, base composition, and codon usage found in other related species. The overall base composition of the mitochondrial genome is 37.0 % A, 32.2 % T, 20.2 % C, and 10.6 % G. All 13 protein-coding genes (PCGs) began with typical ATN initiation codon. Nine of the 13 PCGs have a complete termination codon, but the remaining four genes (COI, COIII, ND5, and ND4) terminate with an incomplete T. Phylogenetic analyses are carried out based on the concatenated dataset of 13 PCGs and two rRNAs of Tettigoniidae species available in GenBank. Both Bayesian inference and Maximum Likelihood analyses recovered each subfamily as a monophyletic group. Regardless of the position of Lipotactinae, the relationships among the subfamilies of Tettigoniidae were as follows: ((((Tettigoniinae, Bradyporinae) Meconematinae) Conocephalinae) Hexacentrinae). The topological structure of the phylogeny trees showed that the Xizicus (Haploxizicus) maculatus is closer to Xizicus (Xizicus) fascipes than Xizicus (Eoxizicus) howardi.

## Introduction

Insect mitochondrial genome (mitogenome) occurs as a small (15–20kb), circular, and double-stranded DNA molecules, including 13 protein-coding genes (PCGs), 22 transfer RNA (tRNA) genes, two ribosomal RNA (rRNA) genes (Boore 1999; Cameron, 2014) and at least one large non-coding region related to the control of replication and transcription (Clayton 1992; Fernandez-Silva et al. 2003). Recently, Next-Generation Sequencing (NGS) technology, combined with bioinformatic annotation, which presently used for generating mitogenomes without using PCR, has led to a rapid increase in the number of sequenced mitogenomes (Cameron 2014). In recent years, more and more mitochondrial entire genomes of Orthoptera have been sequenced (Yang et al. 2016; Zhou et al. 2014; 2017). Up to now, 24 Tettigoniidae (Orthoptera) mitogenomes have been reported or registered in GenBank, including only five Meconematine mitogenomes.

*Xizicus* Gorochov, 1993 is a diverse genus in Meconematinae with 68 species in the world, currently divided into seven subgenera by traditional taxonomy based on comparative morphology: *Xizicus* s. str., *Axizicus*, *Eoxizicus*, *Furcixizicus*, *Haploxizicus*, *Paraxizicus*, *Zangxizicus* according to the OSF website (Cigliano et al. 2018). However, great controversies still exist in the defining characteristics and taxonomic status of these subgenera, and the assignment of some species changes frequently and has undergone numerous taxonomic revisions with contradicting conclusions (Gorochov 1998; Jiao et al. 2013; Wang et al. 2014). Integrative taxonomy combining multiple kinds of data and complementary perspectives (e.g. morphology, ecology and DNA sequences) has been recognized as a particularly efficient means to species delimitation and genus-level classification (Cruz-Barraza et al. 2012; Heneberg et al. 2015; Korshunova et al. 2017). The mitogenome is considered a powerful marker and is extensively applied for resolving metazoan phylogenetic relationships at both deep and shallow taxonomic levels (Cameron 2014; Zhou et al. 2017).

To date, the mitogenomes of Xizicus (Xizicus) fascipes and Xizicus (Eoxizicus) howardi have been sequenced (Yang et al. 2012; Liu 2017). Xizicus (Haploxizicus) maculatus (Xia & Liu, 1993) is a representative species of subgenus Haploxizicus distributed in Hunan of China, which was reported by Xia and Liu (1993) within the genus *Xiphidiopsis*. Later, Gorochov (1998) proposed the genus *Axizicus* and transferred *Xiphidiopsis maculatus* to this new genus. Wang et al. (2014) subsequently transferred it to the new subgenus Haploxizicus mainly based on the typical characteristic of vertex disc with four longitudinal bands and the simple cercus, and this classification viewpoint was adopted by the Orthoptera Species File website (Cigliano et al. 2018). Type locality of X. (H.) maculatus is Cili country of Hunan Province. Specimen used in this study was collected from Hainan Province for the first time. In this study, we provided a thorough description of the complete mitochondrion genome of X. (H.) maculatus, compared the relative synonymous codon usage with other two subgenera species. Additionally, phylogenomic analyses were conducted based on mitochondrial genome data of Tettigoniidae available in GenBank with the purpose of investigating the phylogenetic position of X. (H.) maculatus and better understanding the phylogenetic relationship of Tettigoniidae.

## Materials and methods

### Taxon sampling and sequencing

The specimen of X. (H.) maculatus was collected at Jianfengling in Hainan, China in June 2017 and stored in 100 % ethanol at −4 °C. Genomic DNA was extracted from the leg muscle tissue of a single adult male species of using a DNeasy Blood & Tissue Kit (Qiagen, USA) according to the manufacturer’s instruction and sent to a company for library prep and sequencing (Genesky Biotechnologies Inc., Shanghai). The library was prepared using a TruSeq DNA sample Preparation kit (Vanzyme, China) and sequenced with 150 bp pair-end reads on the Illumina Hiseq 2500 sequencing platform (Illumina, USA).

### De novo assembly and annotation of the Xizicus (Haploxizicus) maculatus mitogenome

13,799,778 raw reads were sequenced by the Illumina Hiseq 2500 platform. The raw paired-end reads were filtered to obtain high-quality clean reads by using CLC Genomics Workbench 8 (CLC Bio, Aarhus, Denmark) with default parameters. Then the filtered reads were aligned to the mitochondrial genome of X. (X.) fascipes (JQ326212) as a reference using MITObim v1.8 (Hahn et al. 2013) and Mira 4.0.2 (Chevreux et al. 2004) to assembly. All of 32,608 clean mitochondrial reads yielded an average coverage of 255.4 X. The complete mitochondrial genome sequence was annotated using the software Geneious v 10.1.2 (Biomatters Ltd., Auckland, New Zealand) by comparing with the mitochondrial genome of X. (X.) fascipes (JQ326212). The tRNA genes were predicted using online software MITOS (Bernt et al. 2013). The nucleotide relative synonymous codon usage (RSCU) values of PCGs were analysed with MEGA v7 software (Kumar et al. 2016).

### Phylogenetic analyses

Phylogenetic analyses were performed on the concatenated datasets of PCGs and rRNAs of the newly sequenced mitogenome and 24 Tettigoniidae species downloaded from GenBank, with two Phaneropteridae taxa (*Ducetia japonica* and *Phyllomimus sinicus*) selected as the outgroups. Alignment of each protein-coding gene inferred from the amino acid alignment was performed using MEGA v7.0 (Kumar et al. 2016), and the alignment results were then concatenated. Bayesian inference (BI) analysis was used for phylogenetic reconstruction with MrBayes 3.1.2 (Ronquist and Huelsenbeck 2003) under the partitioned models chosen by PartitionFinder 2 (Lanfear et al. 2017). In the BI analysis, 10,000,000 generations were run, with four MC chains, and the trees were sampled every 1000 generations with a burn-in step. The confidence values for the BI tree were expressed as the Bayesian posterior probabilities in percentages. A maximum likelihood (ML) tree was constructed using RAxML 8.0 (Stamatakis 2014) and the optimal partitions and best models were also selected by PartitionFinder 2 and the robustness of the phylogenetic results was tested through bootstrap analysis with 1000 replicates in RAxML and the bootstrap support values were printed on the best ML tree.

## Results and discussion

### Genome organization

The complete mitogenome of X. (H.) maculatus is 16,358 bp in length and has been deposited in GenBank under accession no. MG779499. Xizicus (H.) maculatus mtDNA is larger than that observed in other species in Meconematinae, which typically ranged from 16,044 bp (Zhou et al. 2017) to 16,166 bp (Yang et al. 2012).

**Figure 1.**
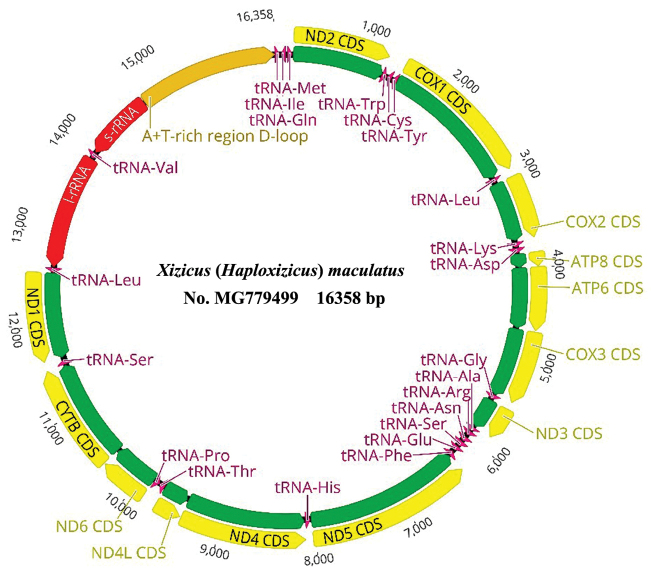
Circular visualization of the mitogenome of Xizicus (Haploxizicus) maculatus.

The mitochondrial genome structure is detailed in Table 1. It contained a typical gene content found in metazoan mitogenomes: 13 protein-coding genes (PCGs), 22 transfer RNA (tRNA) genes, two ribosomal (rRNA) genes, and one control region (Table 1, Fig. 1). Gene order and arrangement was identical to the X. (X.) fascipes mitogenome. The X. (H.) maculatus mitochondrial genes were separated by a total of 65 bp intergenic spacer sequences, which spreaded over ten regions and range in size from one to 17 bp. There were 13 overlaps amongst all 48 bp and the longest overlaps of 8 bp were located between tRNA^Trp^-tRNA^Cys^ and tRNA^Tyr^-COI. The overall base composition of the whole mitochondrial genome was 37.0 % A, 32.2 % T, 20.2 % C, and 10.6 % G, exhibiting obvious anti-G and AT bias (69.2 %) which was slightly lower than X. (X.) fascipes (70.2 %) and X. (E.) howardi (71.0 %).

**Table 1.** Organization of the Xizicus (Haploxizicus) maculatus mitogenome.

Gene/region	Position	Size	Direction	Initiation codon	Termination codon	anticodon
tRNA ^Ile^	1-66(-3)	66	F			GAT
tRNA ^Gln^	64-132(+8)	69	R			TTG
tRNA ^Met^	141-204	64	F			CTA
ND2	205-1233(-2)	1029	F	ATT	TAA	
tRN^ATr^p	1232-1297(-8)	66	F			TCA
tRNA ^Cys^	1290-1353	64	R			GCA
tRNA ^Tyr^	1354-1419(-8)	66	R			GTA
COI	1412-2951	1540	F	ATT	T	
tRNA ^Leu(UUR)^	2452-3016(+3)	65	F			TAA
COII	3020-3703(+1)	684	F	ATG	TAA	
tRNA ^Lys^	3705-3774(-1)	70	F			CTT
tRNA ^Asp^	3774-3839	66	F			GTC
ATP8	3840-4004(-7)	165	F	ATT	TAA	
ATP6	3998-4675(-1)	678	F	ATG	TAA	
COIII	4675-5461	787	F	ATG	T	
tRN^AGl^y	5462-5526	65	F			TCC
ND3	5527-5880(+2)	354	F	ATC	TAA	
tRNA ^Ala^	5883-5946(-1)	64	F			TGC
tR^NAA^rg	5946-6008(+16)	63	F			TCG
tRNA ^Asn^	6025-6090(+2)	66	F			GTT
tRNA ^Ser(AGN)^	6093-6159	67	F			GCT
tRN^AGl^u	6160-6226(+14)	67	F			TTC
tRNA ^Phe^	6305-6421	65	R			GAA
ND5	6306-8037	1732	R	ATT	T	
tRNA ^His^	8038-8101	64	R			GTG
ND4	8102-9440(-7)	1339	R	ATG	T	
ND4L	9434-9730(+1)	297	R	ATG	TAA	
tRNA ^Thr^	9732-9802(-1)	71	F			TGT
tRNA ^Pro^	9802-9867(+1)	66	R			TGG
ND6	9869-10396(-1)	528	F	ATA	TAA	
Cytb	10396-11532(-2)	1137	F	ATG	TAG	
tRNA ^Ser(UCN)^	11531-11599(+17)	69	F			TGA
ND1	11617-12570(-6)	954	R	ATA	TAG	
tRNA ^Leu(CUN)^	12565-12628	64	R			TAG
lrRNA	12629-13932	1304	R			
tRNA ^Val^	13933-14003	71	R			TAC
srRNA	14004-14788	785	R			
Control region	14789-16358	1570	F			

### Protein-coding genes

The total length of all 13 PCGs was 11,224 bp, and the overall A+T content of X. (H.) maculatus PCGs was 68.3 %. The initiation codons of all PCGs were typical with ATN (COII, ATP6, COIII, ND4, ND4L, and Cytb with ATG, ND2, COI, ATP8, ND5 with ATT; ND6, ND1 with ATA, Only ND3 with ATC). The ATN codon was prevalently regarded as initiation codons for mitogenome PCGs in insects. Seven genes (ND2, COII, ATP8, ATP6, ND3, ND4L, ND6) used TAA as the termination codons, and two genes (Cytb, ND1) were stopped with TAG. COI, COII, ND5, and ND4 had an incomplete termination codon T (Table 1). The incomplete termination codon is common in metazoan mitochondrial genomes and exhibit function after post-transcription polyadenylation converts into full stop codon (Ojala et al. 1981).

**Figure 2.**
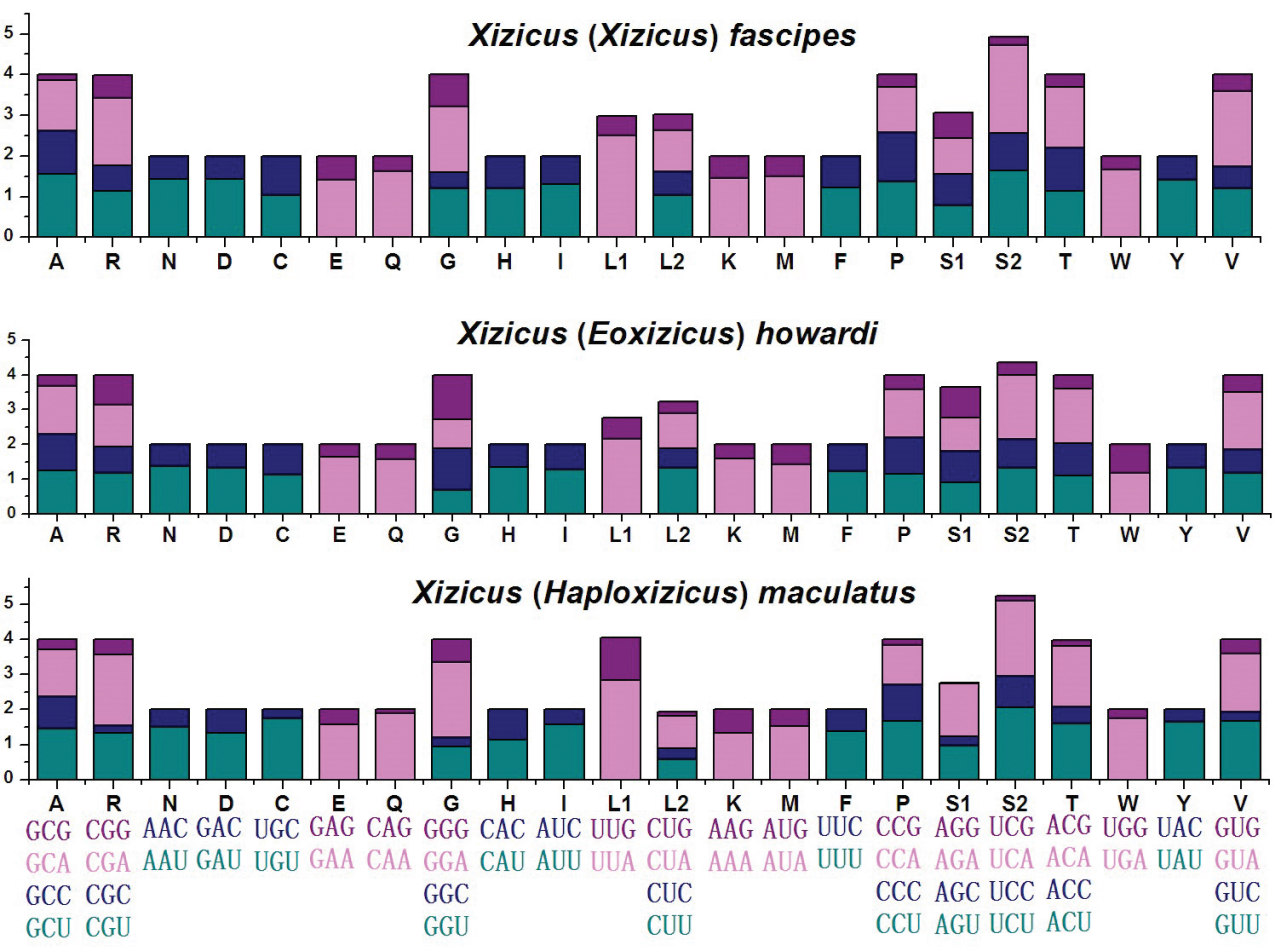
Relative synonymous codon usage of X. (X.) fascipes, X. (E.) howardi, X. (H.) maculatus mitochondrial protein-coding genes. Condon families are provided on the x-axis.

The four most-used amino acids in X. (H.) maculatus were Leu (16.1 %), Ser (8.9 %), Phe (8.7 %), and Ile (8.4 %), whose proportions were similar to those observed in other Tettigoniidae species. All codons were present in the protein-coding genes of this mitogenome. Excluding incomplete termination codons, there were 3,735 codons in the X. (H.) maculatus protein-coding genes. The codon usage in X. (H.) maculatus appeared to be typical of other insect mitochondrial sequences. The RSCU analysis indicated that codons including A or T at the third position were always overused compared with other synonymous codons in *Xizicus* (Fig. 2). The codon usage could also reflect nucleotide bias.

### Ribosomal and transfer RNA genes

The length of tRNA genes ranged from 63 to 71 bp and the relative locations for each tRNA are shown in Table 1. All tRNA genes had the typical cloverleaf secondary structures except for tRNA^Ser(AGN)^. The secondary structures of tRNA^Ser(AGN)^ was completely identical with X. (X.) fascipes and showed a lengthened anticodon stem (9 bp) with a bulging nucleotide in the middle, an unusual 6 bp length T-stem, a mini DHU arm (2 bp), and no connector nucleotides (Yang et al. 2012).

The lrRNA and srRNA were 1304bp and 785bp in length, respectively. They were located between tRNA^Leu(CUN)^ and A+T-rich region, being separated by tRNA^Val^.

### Non-coding regions

The control region was 1570 bp in length and located between srRNA and tRNA^Ile^ in X. (H.) maculatus mitogenome, and was composed of 64.4 % A and T nucleotides (Fig. 1; Table 1). The control region is characterized by a high AT content and is thought to be involved in the regulation of mtDNA transcription and replication (Bae et al., 2004), whose size differences are not only because of high rates of nucleotide substitution, insertion or deletion, but also due to the length of tandem repeat unit and the number of tandem repetitions (Yang et al. 2012). The sequence analysis revealed four tandem repeats size from 26 to 162 bp, contributing 738 bp to the length of the region.

### Phylogenetic relationships

**Figure 3.**
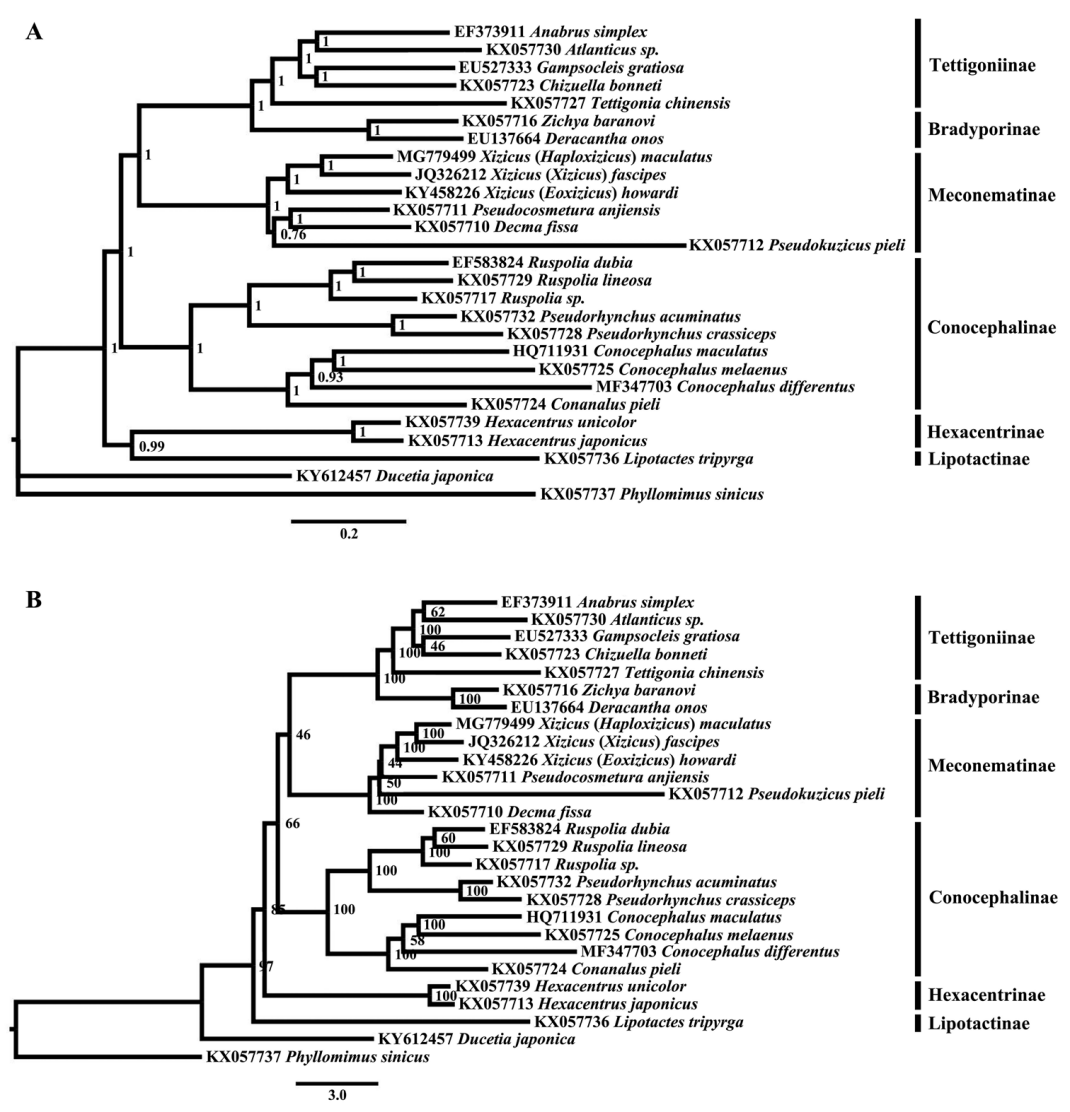
Phylogenetic reconstruction of Tettigoniidea using mitochondrial PCGs and rRNA concatenated dataset. **A** Bayesian result, applicable posterior probability values are shown **B** Maximum likelihood result with applicable bootstrap values shown.

Bayesian analyses and maximum likelihood produced identical topologies using the best-fit partitioning scheme and site-homogeneous models, excepting the location of *Lipotactes tripyrga* (Lipotactinae) and genera relationships in Meconematinae (Fig. 3). Bayesian inference recovered each Tettigoniinae, Bradyporinae, Conocephalinae, and Meconematinae as a monophyletic group with strongly supported (PP ≥ 0.93), while the monophyly of Conocephalinae and Meconematinae (only include the tribe Meconematini) was not well supported in ML topology. The position of *L. tripyrga* was at basal in the ML tree while it formed the most basal clade together with Hexacentrinae in the BI analysis. The relationship of Lipotactinae with other subfamilies was unascertainable may due to only one taxa used in analyses.

In present study, regardless of the position of Lipotactinae, the relationships among the subfamilies of Tettigoniidae were as follows: ((((Tettigoniinae, Bradyporinae) Meconematinae) Conocephalinae) Hexacentrinae), which was congruent with the phylogenetic results using site-homogeneous both in ML and BI analyses (Zhou et al. 2017). The relationships among subfamilies within Tettigoniidae were sensitive to the methods used for tree reconstruction and the molecular maker used. Previous phylogenetic studies based on multi-molecular makers (28SrDNA, 18SrDNA, COII, Histone and Wingless genes) inferred a striking difference results that placed Hexacentrinae and Meconematinae as more ‘advanced’ groups sister to the clade consisting of Tettigoniinae and Lipotactinae, and Conocephalinae as the more ‘primitive’ group diverging at an earlier node (Mugleston et al. 2013). The present topologies placed Tettigoniinae as an apical node sister to Bradyporinae and then assembled with Meconematinae, which was congruent with the phylogenetic results using site-homogeneous both in ML and BI analyses (Zhou et al. 2017), while differed with the site-heterogeneous CAT-GTR model tree (Zhou et al. 2017). Recent studies suggest that analysis model used in mitochondrial phylogenetic reconstruction potentially impact phylogenies result when genomic data existing lineage compositional heterogeneity and saturation due to accelerated substitution rates causing homoplasy (Li et al. 2015; Zhou et al. 2017).

The generic relationships within the subfamily Meconematinae were not identical in the BI and ML trees. The relationships between the four genera were as follows: (((*Pseudocosmetura*, *Decma*) *Pseudokuzicus*) *Xizicus*) in the BI tree (Fig. 3A); (((*Xizicus*, *Pseudokuzicus*) *Pseudocosmetura*) *Decma*) in the ML tree (Fig. 3B). Xizicus (H.) maculatus, X. (X.) fascipes and X. (E.) howardi clustered into one clade in both analyses, and X. (H.) maculatus was closer to the nominate species X. (X.) fascipes than X. (E.) howardi. Additional taxon sampling will be needed to clarify the relationships of genera in Meconematinae.
